# Association between acupuncture exposure and mortality in people with disabilities diagnosed with ischemic heart disease: a nationwide retrospective cohort study in the Republic of Korea

**DOI:** 10.1186/s12906-026-05434-y

**Published:** 2026-06-12

**Authors:** Hyungsun Jun, Jae-Uk Sul, Moon Joo Cheong, Hae‑Rim Kim, Inae Youn, Ye-Seul Lee, Jungtae Leem

**Affiliations:** 1 Department of Diagnostics, College of Korean Medicine, Dongshin University, Naju, 58245 Republic of Korea; 2 Korean Medicine Convergence Research Institute, College of Korean Medicine, Dongshin University, Naju, 58245 Republic of Korea; 3 Dongshin University Gwangju Korean Medicine Hospital, Gwangju, 61619 Republic of Korea; 4 Department of Medical Counseling, College of Health Science, Wonkwang University, Iksan, 54538 Republic of Korea; 5 Department of Statistical Data Science, University of Seoul, Seoul, 02504 Republic of Korea; 6 Department of Acupuncture & Moxibustion, National Medical Center, Seoul, 04564 Republic of Korea; 7 Jaseng Spine and Joint Research Institute, Jaseng Medical Foundation, Gangnamdaero 540, Seoul, 06110 Republic of Korea; 8 Department of Diagnostics, College of Korean Medicine, Wonkwang University, Iksan, 54538 Republic of Korea; 9 Department of Il-won Integrated Medicine, Wonkwang University Korean Medicine Hospital, Iksan, 54538 Republic of Korea; 10 Research Center of Traditional Korean Medicine, College of Korean Medicine, Wonkwang University, Iksan, 54538 Republic of Korea; 11 College of Korean Medicine, Wonkwang University, 460, Iksan-daero, Sin- dong, Iksan, 54538 Jeollabuk-do Republic of Korea

**Keywords:** Acupuncture, People with disability, Ischemic heart disease, Mortality, Retrospective cohort study

## Abstract

**Background:**

People with disabilities (PWDs) have higher incidence and mortality rates of cardiovascular disease than non-PWDs. Considering the mutual influence of cardiovascular diseases and disabilities, careful observation and complementary management beyond standard treatments are essential when ischemic heart disease (IHD) occurs in PWDs. This study investigated the association between acupuncture treatment and reduced mortality in PWDs diagnosed with IHD.

**Methods:**

Using a database from the National Health Insurance Service of Korea, PWDs newly diagnosed with IHD were included in this study. Exposure to acupuncture treatment within one year after diagnosis was used to classify participants into the exposure and non-exposure groups, with follow-up for three years or until death. Propensity score matching (PSM) was used to adjust for group imbalances, and survival analysis was conducted using the Cox proportional hazards model to calculate hazard ratios (HRs) and 95% confidence intervals (CIs) for mortality. Dose-response and subgroup analyses were also performed.

**Results:**

After PSM, 38,157 PWDs were included in each group. The acupuncture group showed a significantly lower hazard of mortality, approximately 22% lower than that of the control group (adjusted HR 0.78, 95% CI 0.74–0.81). In the dose-response analysis, the group receiving the highest dose of acupuncture demonstrated the lowest hazard of mortality (adjusted HR 0.67, 95% CI 0.62–0.72). Most subgroups also showed a reduced mortality hazard in the acupuncture-treated groups, with the highest dose group exhibiting the lowest hazard.

**Conclusion:**

This study is a foundational investigation suggesting that acupuncture treatment is associated with reduced mortality in PWDs diagnosed with IHD. Although future research is needed to establish causality, acupuncture should be considered a beneficial option for managing IHD in PWDs.

**Supplementary Information:**

The online version contains supplementary material available at 10.1186/s12906-026-05434-y.

## Background

According to the World Bank, approximately 15% of the global population has some form of disability [1]. In South Korea, as of 2020, approximately 2.6 million people were registered with disabilities (PWDs), accounting for approximately 5% of the total population [2]. PWDs have a shorter average life expectancy and more comorbid diseases than non-PWDs [3] and are also known to have a poorer quality of life [4]. Owing to various physical and psychological comorbidities, the medical costs for PWDs are higher than those for non-PWDs [5]. Although PWDs visit medical institutions more frequently, their unmet medical needs are greater than those of non-PWDs [6]. Therefore, effective medical and healthcare approaches are needed to improve the health status of PWDs.

Ischemic heart disease (IHD), a leading cause of death among cardiovascular diseases, significantly increases the burden of disease, mortality, catastrophic health expenditure, and serious sequelae, such as heart failure [7]. Disability-adjusted life years (DALYs) due to cardiovascular disease have been continuously increasing worldwide, with the total number of DALYs due to IHD rising steadily since 1990, reaching 182 million (95% uncertainty interval: 170–194 million) in 2019 [8]. This indicates that cardiovascular disease is a significant factor that causes and exacerbates disabilities. PWDs have higher incidence and mortality rates of cardiovascular disease than non-PWDs [9], with reports indicating that those with physical disabilities are at an even higher risk of coronary heart disease [10]. Disability and cardiovascular disease influence each other [11], necessitating the careful management of cardiovascular diseases in PWDs. Antiplatelet drug treatment is the first choice for the management and prevention of IHD [12]. However, IHD can recur despite antiplatelet therapy, and its recurrence rate increases significantly with age [13]. For high-risk patients who are not effectively managed with drug therapy, stent insertion via percutaneous coronary intervention or coronary artery bypass surgery may be performed [14]. However, recurrence after revascularization remains a therapeutic challenge that threatens the physical and mental health of patients with IHD [15]. Despite conventional management, the mortality rate associated with IHD is higher in PWDs than in non-PWDs, and there are insufficient therapeutic options and clinical evidence regarding the management and prevention of IHD in PWDs. Therefore, a complementary and integrative strategy is required to effectively manage IHD in PWDs.

Acupuncture is widely used to treat IHD in East Asia. In Korea and Taiwan, acupuncture is covered by public health insurance, which allows the extensive use of health insurance claims data for acupuncture clinical research. A previous nationwide cohort study using the Taiwan database found that the hazard ratio (HR) for myocardial infarction in stroke patients who underwent acupuncture treatment was 0.86 (95% confidence interval [CI], 0.80 to 0.93) compared to the non-acupuncture group [16]. Another study demonstrated a dose-response relationship, suggesting a likely causal relationship [17]. A recent retrospective cohort study using a Korean database indicated that acupuncture treatment reduced the incidence of major adverse cardiac events, including mortality, myocardial infarction, and stroke, in patients with hypertension [18]. Additionally, a randomized controlled trial (RCT) showed a significant reduction in the angina attack rate in an acupuncture treatment group [19]. Studies have also been conducted on the mechanisms of action underlying the efficacy of acupuncture in IHD. These studies suggest that the primary mechanisms of acupuncture include increasing the maximum workload, suppressing blood pressure, and regulating the sympathoexcitatory reflex [20, 21]. These mechanistic and efficacy studies indicate that acupuncture can be an effective therapeutic option to overcome the limitations of conventional medicine in the treatment of IHD.

However, these studies did not focus on PWDs and were conducted in non-PWD populations. Although acupuncture is widely used in clinical practice to treat PWDs, evidence from clinical studies is insufficient. To effectively apply acupuncture to PWDs, a study on the effectiveness of acupuncture treatment for IHD in this population is necessary. Recently, exploratory research using real-world data has been actively conducted to develop research hypotheses and study designs before conducting confirmatory RCTs. In particular, the Korean National Health Insurance Service (NHIS) data offer the advantage of overcoming some of the limitations of observational research because it is a single-payer healthcare system for the entire nation [22]. Although epidemiological studies have been conducted on the incidence and mortality of cardiovascular diseases in PWDs [9], no RCTs or cohort studies have examined the association between acupuncture treatment and the clinical prognosis of IHD in PWDs. Conducting RCTs on PWDs presents more practical obstacles than conducting research on non-PWDs. Therefore, this study aimed to conduct a retrospective cohort study using NHIS claims data in Korea to determine whether acupuncture treatment within one year of diagnosis affects mortality in PWDs newly diagnosed with IHD. Additionally, the study will analyze the dose-response relationship according to the number of acupuncture treatment sessions to explore the potential causal relationship.

## Methods

### Data source

This retrospective nationwide cohort study was conducted using data from the Korean NHIS claims database. Nearly all Koreans (97%) are enrolled in the national health insurance, while the remaining 2.8% of the population, comprising low-income families, are covered by Medicaid. As the NHIS database also includes Medicaid, it represents the entire Korean population. In addition, all Korean medical institutions are covered by the NHIS single-payer system [22]. The NHIS data include various sociodemographic information such as age, sex, comorbidities, income, residence area, and severity of disability. It also contains comprehensive treatment-related data, including medical expenses, primary and secondary diagnoses, treatment dates and durations, hospitalization days, prescribed medications and durations, medical examinations, and treatment interventions [22]. These data can be linked to death records from the National Statistical Office of Korea. The metadata and related materials regarding NHIS claims data are publicly available on the National Health Insurance Data Sharing Service website (http://nhiss.nhis.or.kr). Researchers can apply for specific research data from the NHIS source database, which is approved by the NHIS Inquiry Committee of Research Support. For this study, total health insurance claims data from 2013 to 2020 for registered PWDs in Korea (NHIS-2021-1-301) were provided to authorized researchers who accessed the data for analysis using cloud services in a designated location, adhering to strict privacy policies [23]. This study was approved by the Institutional Review Board of Dongshin University Korean Medicine Hospital (DSGOH_E_2021_003) and was conducted in accordance with the principles of the Declaration of Helsinki.

### Study population

**Fig. 1:**
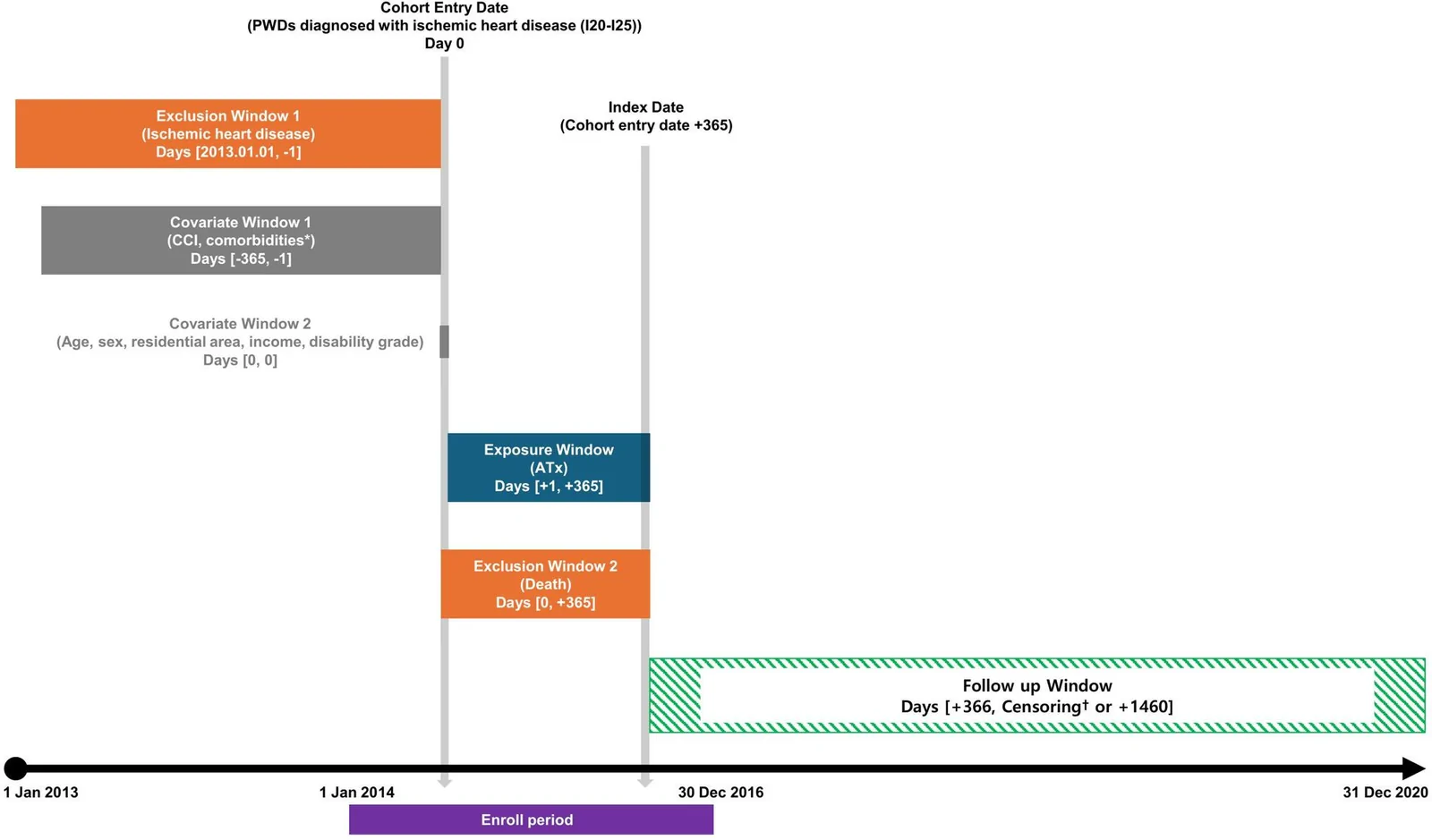
Study design. ATx, acupuncture treatment; CCI, Charlson Comorbidity Index; PWDs, people with disabilities. *Prespecified comorbidities are hypertension, diabetes mellitus, dyslipidemia, ischemic stroke, chronic obstructive pulmonary disease, atrial fibrillation, peripheral artery disease, chronic kidney disease, and chronic liver disease. †Censored at the earliest occurrence of death or the end of the study period. Between January 2014 and December 2016, individuals with mild or severe disabilities who were newly diagnosed with ischemic heart disease were included in this study. The day of diagnosis was designated as the cohort entry date, and one year later was set as the index date, during which acupuncture exposure was assessed. From the index date onward, participants were followed for up to three years to monitor mortality.

The study design and scheme are illustrated in Fig. 1 [24]. The study cohort was based on NHIS claims data for PWDs from 2013 to 2020. Individuals can be registered as PWDs upon receiving a disability diagnosis from a medical institution, and these records are incorporated into the NHIS database via the Korea Social Security Information Service. According to NHIS data, disability ratings of 1–2 are classified as severe disabilities, whereas ratings of 3–6 are classified as mild disabilities. In this study, individuals who met the criteria for mild or severe disability were defined as PWDs and included as participants. IHD was defined according to the International Classification of Diseases, 10th Revision (ICD-10) codes I20, I21, I22, I23, I24, and I25. IHD was diagnosed according to the Health Insurance Review and Assessment Service standard classification guidelines for diseases and intervention [25]. Only newly diagnosed IHD cases from January 1, 2014, to December 31, 2016, were included. Previously diagnosed IHD cases were excluded using 2013 as the washout period. IHD was identified in PWDs aged ≥ 19 years. In this study, the landmark was set as one year after IHD diagnosis; therefore, only participants who survived for more than one year after their first diagnosis of IHD were included. The appropriate landmark time may vary by disease, and an unsuitable time point can potentially lead to an overestimation of study results. Conversely, extending the landmark time tends to reduce bias and thereby lower the risk of overestimation [26–28]. Considering these factors and referring to previous cohort studies [18, 29], this study applied a one-year landmark time.

The acupuncture-exposed group was identified using codes 40,011 (acupuncture, single body part) and 40,012 (acupuncture, multiple body parts) [30]. The acupuncture group was defined as those who received acupuncture at least twice within 1 year of being diagnosed with IHD, according to previous acupuncture studies using the NHIS database [18, 31]. The non-exposure group (control group) included PWDs who did not receive acupuncture treatment during the 1 year after the first diagnosis of IHD.

### Clinical outcomes and covariates

The primary outcome measure was all-cause mortality. The date of death was confirmed by combining the national population death registration data from the National Statistical Office with a unique personal identification number [32]. The disease NHIS claim codes are based on operational definitions validated in previous studies [33]. The cohort entry date was the day of the first IHD diagnosis. Mortality was monitored from the landmark point (1 year after the first IHD diagnosis) to the date of death or the end of the study (4 years after the first IHD diagnosis). Covariates included socioeconomic variables such as age, sex, residential area, income, and severity of disability. General health conditions were indirectly evaluated using the Charlson Comorbidity Index (CCI). The CCI score was calculated based on the time point within one year immediately before the new diagnosis of IHD. CCI is a frequently used statistic that accounts for 19 concurrent disorders in medical data [34]. Detailed definitions of each clinical variable, covariate, and population are provided in Additional File 1.

### Statistical analysis

The baseline characteristics of the acupuncture and control groups were compared using independent t-tests and chi-square tests. Imbalances in baseline characteristics between the two groups were mitigated using propensity score matching (PSM). Propensity scores for being assigned to the acupuncture and control groups were computed using a multiple logistic regression model that considered baseline factors such as age, sex, CCI, disability severity, comorbidities (including hypertension, diabetes mellitus, dyslipidemia, ischemic stroke, chronic obstructive pulmonary disease [COPD], atrial fibrillation, peripheral artery disease, chronic kidney disease, and chronic liver disease), income, and area of residence. Following the calculation of propensity scores, patients were matched using a nearest-neighbor algorithm within a caliper width of 0.01 and a ratio of 1:1 [35]. The standardized mean difference (SMD) was used as the primary metric to assess the balance between the groups. SMD was calculated as the difference in the means of a covariate between groups divided by the standard deviation of the treated group [36]. According to the guidelines, SMD values of 0.1 or 0.25 are acceptable cut-off values for standardized biases [37], with values less than 0.1 indicating insignificant differences between groups. Schoenfeld residuals were used to avoid bias due to the disproportionate severity between the acupuncture and control groups and to confirm the assumption of proportional hazards.

The incidence rate (IR) of all-cause mortality was calculated by dividing the number of incident cases by the person-time at risk per 1000 person-years. HRs and 95% CIs for all-cause mortality were calculated using the Cox proportional hazards model, with the control group as the reference group. Kaplan–Meier curves were used to visualize event-free outcomes in the acupuncture and control groups. To explore the dose-response relationship according to the number of acupuncture treatments, the acupuncture group was divided into four quartiles: Q1 (lowest number of acupuncture sessions) and Q4 (highest number of acupuncture sessions). Additionally, subgroup analyses of the dose-response relationship were conducted according to the covariates used in the survival analysis. Participants were divided into two or three groups based on covariates such as age (< 60, 60–74, ≥ 75), sex (male, female), disability severity (mild, severe), income (low, medium, high), residence (urban, rural), CCI (< 3, ≥ 3), hypertension, diabetes mellitus, atrial fibrillation, chronic kidney disease, chronic liver disease, peripheral artery disease, COPD, and ischemic stroke. Statistical significance was set at *p* < 0.05. SAS version 9.4 (SAS Institute Inc., Cary, NC, USA) and R version 3.3.2 (The R Foundation, www.R-project.org) were used for statistical analysis. The “survival,” “survminer,” “moonBook,” “ggplot2,” “survey,” “optmatch,” and “MatchIt” packages in R software were used for analysis, visualization, and PSM.

## Results

### Patient characteristics

**Fig. 2:**
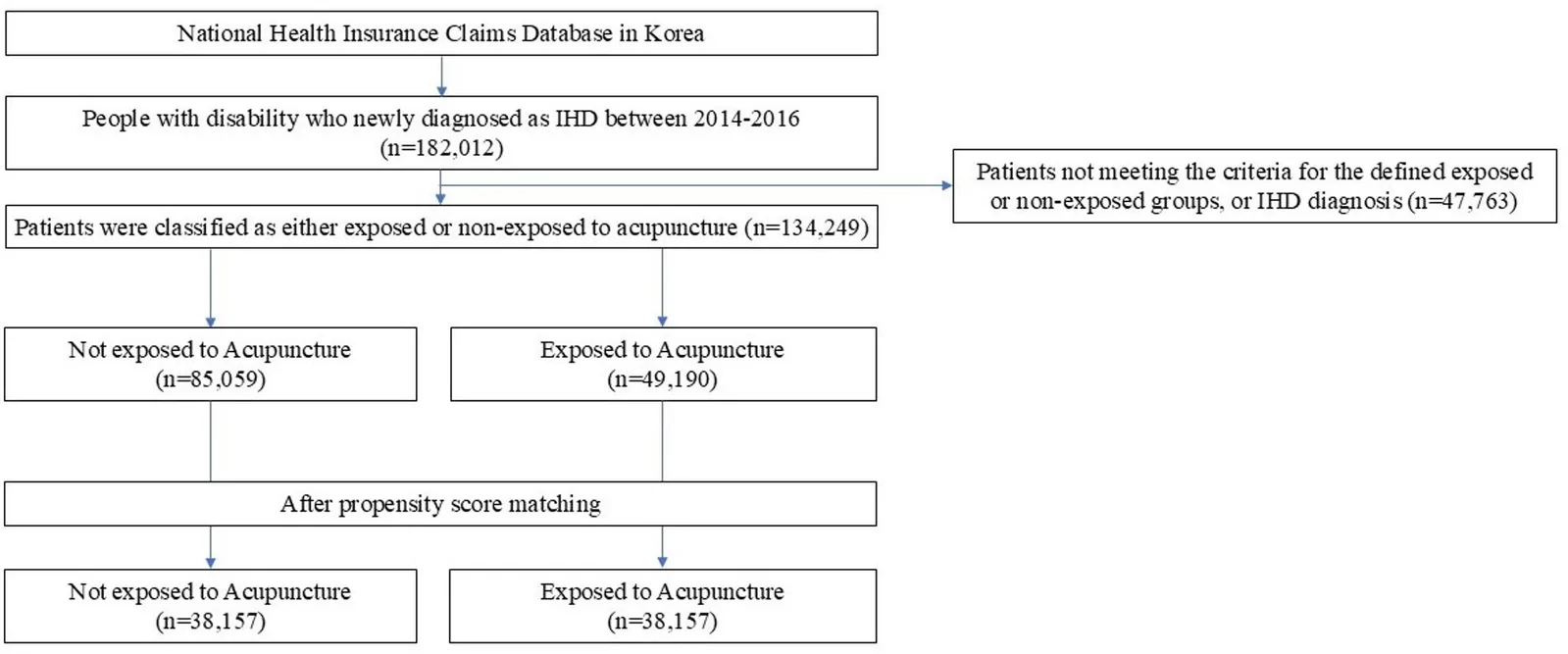
Study flowchart and cohort selection processIHD, ischemic heart disease

From the NHIS database, 182,012 PWDs aged ≥ 19 years who were newly diagnosed with IHD between 2014 and 2016 were identified. Based on the inclusion and exclusion criteria, 49,190 and 85,059 patients were included in the acupuncture and control groups, respectively, before PSM. After PSM, 38,157 patients were analyzed in each group **(**Fig. 2**)**. Before PSM, the acupuncture group had a higher mean age than the control group (69.39 ± 10.77 vs. 67.69 ± 12.91) and greater proportions of women (56.2% vs. 39.9%), as well as urban and rural residents (urban: 19.5% vs. 17.8%; rural: 41.8% vs. 40.5%). The acupuncture group also had higher proportions of high-income individuals (39.6% vs. 35.7%), mild disabilities (87.4% vs. 77.7%), and those with a CCI of 3 or more (31.7% vs. 28.6%). In addition, more patients in the acupuncture group had hypertension, dyslipidemia, ischemic stroke, and COPD than in the control group, whereas fewer had chronic kidney disease (4.5% vs. 10.0%). After PSM, the baseline characteristics were similarly distributed between the two groups **(**Table 1**)**.

**Table 1:** Baseline characteristics before and after propensity score matching

	Before propensity score matching				After propensity score matching			
	Acupuncture (*n*=49,190)	control (*n*=85,059)	*p*	SMD	Acupuncture (*n*=38,157)	Control (n=38,157)	*p*	SMD
Age, years	69.39±10.77	67.69±12.91	<0.001	0.243	69.17±11.10	69.34±11.54	1	<0.001
20–29	83 (0.2)	407 (0.5)			38 (0.1)	38 (0.1)		
30–39	414 (0.8)	1595 (1.9)			278 (0.7)	278 (0.7)		
40–49	1883 (3.8)	5732 (6.7)			1395 (3.7)	1395 (3.7)		
50–59	7266 (14.8)	16201 (19.0)			5824 (15.3)	5824 (15.3)		
60–69	13520 (27.5)	23303 (27.4)			10640 (27.9)	10640 (27.9)		
70–79	18856 (38.3)	25461 (29.9)			14424 (37.8)	14424 (37.8)		
≥80	7168 (14.6)	12360 (14.5)			5558 (14.6)	5558 (14.6)		
Sex			<0.001	0.329			0.913	0.001
Male	21556 (43.8)	51085 (60.1)			17603 (46.1)	17619 (46.2)		
Female	27634 (56.2)	33974 (39.9)			20554 (53.9)	20538 (53.8)		
Residential Area			<0.001	0.063			1	<0.001
Metropolitan	18997 (38.8)	35337 (41.7)			15257 (40.0)	15257 (40.0)		
Urban	9548 (19.5)	15115 (17.8)			6598 (17.3)	6598 (17.3)		
Rural	20462 (41.8)	34343 (40.5)			16302 (42.7)	16302 (42.7)		
Income			<0.001	0.095			1	<0.001
Medical aid	7259 (14.8)	14937 (17.6)			5099 (13.4)	5099 (13.4)		
Low	10101 (20.5)	17745 (20.9)			7667 (20.1)	7667 (20.1)		
Middle	12362 (25.1)	21972 (25.8)			9734 (25.5)	9734 (25.5)		
High	19468 (39.6)	30405 (35.7)			15657 (41.0)	15657 (41.0)		
Severity of disability			<0.001	0.257			0.592	0.004
Severe	6195 (12.6)	18944 (22.3)			4117 (10.8)	4164 (10.9)		
Mild	42995 (87.4)	66115 (77.7)			34040 (89.2)	33993 (89.1)		
CCI score			<0.001	0.125			1	<0.001
0	8312 (16.9)	17959 (21.2)			7867 (20.6)	7867 (20.6)		
1	13772 (28.0)	24437 (28.9)			12101 (31.7)	12101 (31.7)		
2	11502 (23.4)	18317 (21.7)			9084 (23.8)	9084 (23.8)		
≥3	15604 (31.7)	24346 (28.6)			9105 (23.9)	9105 (23.9)		
Hypertension	30965 (63.0)	50177 (59.4)	<0.001	0.075	24063 (63.1)	24180 (63.4)	0.384	0.006
DM	12478 (25.4)	21434 (25.4)	0.861	0.001	8076 (21.2)	8086 (21.2)	0.936	0.001
Dyslipidemia	11686 (23.8)	17370 (20.5)	<0.001	0.078	7396 (19.4)	7342 (19.2)	0.627	0.004
Ischemic stroke	5022 (10.2)	7769 (9.2)	<0.001	0.035	2738 (7.2)	2719 (7.1)	0.8	0.002
COPD	4808 (9.8)	7143 (8.5)	<0.001	0.046	2497 (6.5)	2497 (6.5)	1	<0.001
AF	1523 (3.1)	2746 (3.2)	0.142	0.008	649 (1.7)	630 (1.7)	0.612	0.004
PAD	857 (1.7)	1553 (1.8)	0.228	0.007	158 (0.4)	158 (0.4)	1	<0.001
CKD	2191 (4.5)	8432 (10.0)	<0.001	0.214	1202 (3.2)	1139 (3.0)	0.193	0.01
CLD	2037 (4.1)	3458 (4.1)	0.630	0.003	688 (1.8)	688 (1.8)	1	<0.001

Baseline characteristics are presented as *n* (%) or mean ± SD

*AF* atrial fibrillation, *CCI * Charlson Comorbidity Index, *CKD * chronic kidney disease, *CLD * chronic liver disease, *COPD * chronic obstructive pulmonary disease, *DM * diabetes mellitus, *PAD * peripheral artery disease, *SMD * standardized mean difference

### All-cause mortality

**Fig. 3:**
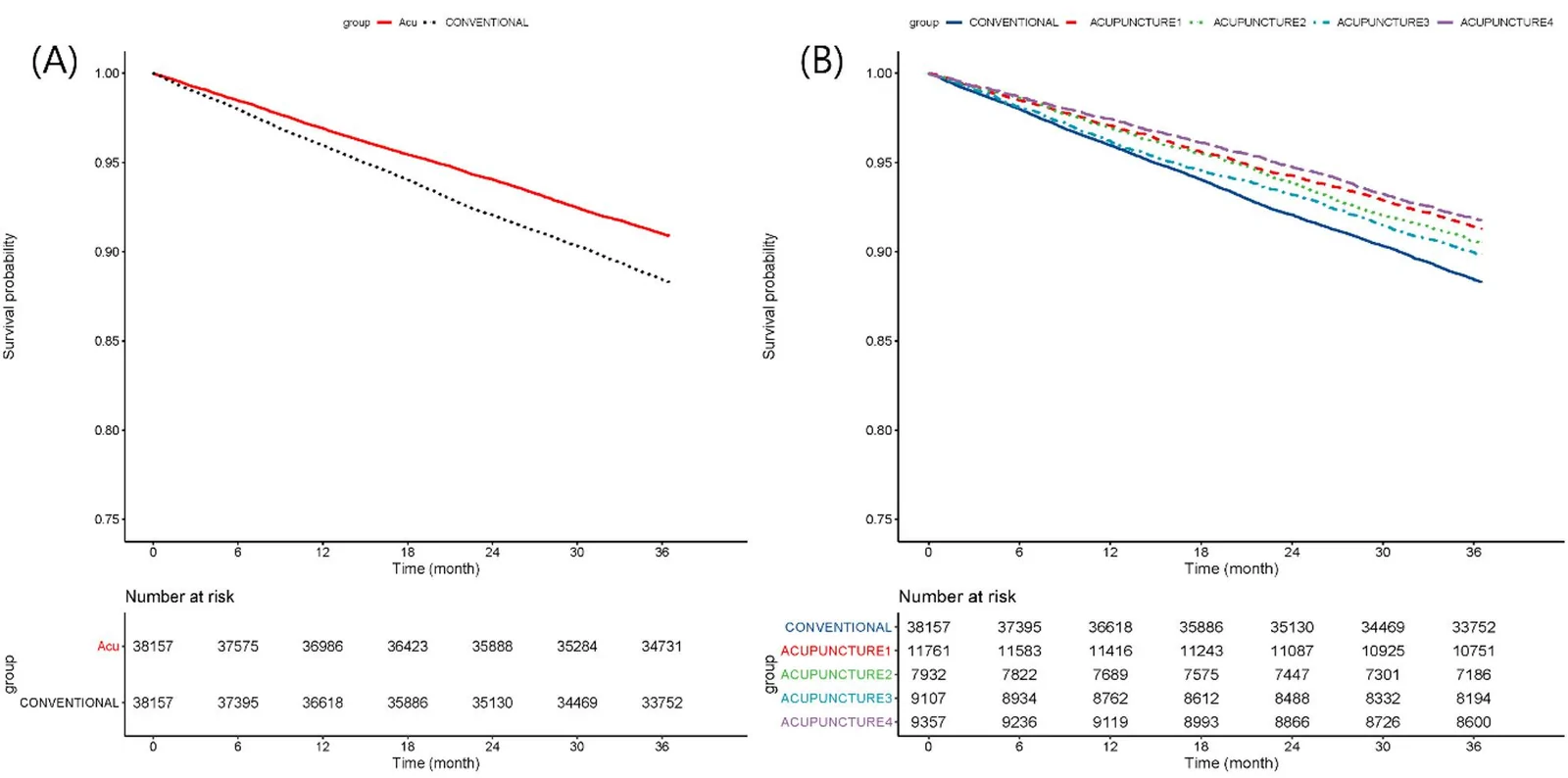
Kaplan–Meier curve of mortality and dose-response relationship. **A** mortality and (**B**) mortality according to acupuncture dose. The conventional group included individuals who had never received acupuncture treatment, whereas the acupuncture group consisted of those who had undergone acupuncture at least twice within one year of being diagnosed with ischemic heart disease. The acupuncture group was further divided into four subgroups based on the number of treatments: acupuncture 1 group (2 to 4 sessions), acupuncture 2 group (5 to 8 sessions), acupuncture 3 group (9 to 18 sessions), and acupuncture 4 group (≥19 sessions)

Before PSM, the acupuncture group was associated with a reduction in all-cause mortality compared to the control group (IR 35.07 vs. 49.77 events per 1000 person-years; adjusted HR 0.76, 95% CI 0.73 to 0.78). After PSM, the acupuncture group remained associated with a reduction in all-cause mortality compared to the control group (IR 31.83 vs. 41.50 events per 1000 person-years; adjusted HR 0.78, 95% CI 0.74 to 0.81) (Table 2). The all-cause mortality rates of the acupuncture and control groups are shown in the Kaplan–Meier survival curve (Fig. 3a).

**Table 2:** Incidence rates and hazard ratios of mortality in acupuncture-exposed and non-exposed groups

	Total, *n*	Events, *n* (%)	IR/1000 person-years	Crude HR (95% CI)	Adjusted HR (95% CI)*
Before PSM					
Control	85,059	11,789 (13.86)	49.77	1 [Ref]	1 [Ref]
Acupuncture	49,190	4,913 (9.99)	35.07	0.70 (0.68**–**0.73)	0.76 (0.73**–**0.78)
After PSM					
Control	38,157	4,465 (11.70)	41.50	1 [Ref]	1 [Ref]
Acupuncture	38,157	3,478 (9.11)	31.84	0.77 (0.73**–**0.80)	0.78 (0.74**–**0.81)

*CI * confidence interval, *HR * hazard ratio, *IR * incidence rate, *PSM * propensity score matching

^*^Adjusted for age, sex, disability severity, income, residence area, hypertension, dyslipidemia, and Charlson Comorbidity Index

### Dose-response relationship of acupuncture with mortality

To explore the dose-response relationship between the number of acupuncture sessions and clinical outcomes, the acupuncture group was divided into four dosage groups based on the number of sessions: acupuncture 1 (2 to 4 sessions, Q1), acupuncture 2 (5 to 8 sessions, Q2), acupuncture 3 (9 to 18 sessions, Q3), and acupuncture 4 (≥ 19 sessions, Q4). In all-cause mortality, the adjusted HR was 0.78 (95% CI 0.73 to 0.84) in the acupuncture 1 group, but it decreased to 0.67 (95% CI 0.62 to 0.72) in the acupuncture 4 group **(**Table 3**)**. All-cause mortality according to dosage is shown in the Kaplan–Meier survival curve **(**Fig. 3b**).** In the dose-response subgroup analysis, the HR for the acupuncture group was significantly lower than 1 in most subgroups; however, in subgroups with atrial fibrillation, chronic kidney disease, and chronic liver disease, the HR for some acupuncture groups was higher than 1. The acupuncture 4 group, which received the highest dose, consistently showed the lowest HR, except in the subgroups of males and those with chronic liver disease, peripheral artery disease, and COPD. Forest plots for subgroup analysis are presented in Additional File 2.

**Table 3:** Incidence rates and hazard ratios of mortality by acupuncture treatment dose

	Acupuncture sessions, *n*	Total, *n*	Events, *n* (%)	IR/1000 person-years	Crude HR (95%CI)	Adjusted HR (95% CI)^*^
Control	0	38,157	4465 (11.70)	41.5	1 [Ref]	1 [Ref]
Acupuncture						
Acupuncture 1	2**–**4	11,761	1028 (8.74)	30.47	0.73 (0.69**–**0.79)	0.78 (0.73**–**0.84)
Acupuncture 2	5**–**8	7932	754 (9.51)	33.25	0.80 (0.74**–**0.87)	0.82 (0.76**–**0.88)
Acupuncture 3	9**–**18	9107	924 (10.15)	35.72	0.86 (0.80**–**0.92)	0.85 (0.79**–**0.91)
Acupuncture 4	≥ 19	9357	772 (8.25)	28.66	0.69 (0.64**–**0.75)	0.67 (0.62**–**0.72)

*CI * confidence interval, *HR* hazard ratio, *IR * incidence rate

^*^Adjusted for age, sex, disability severity, income, residence area, hypertension, dyslipidemia, and Charlson Comorbidity Index

## Discussion

This retrospective cohort study was conducted using survival analysis and the Cox proportional hazards model after PSM. The acupuncture exposed and non-exposed groups were comparable after PSM, with 38,157 participants in each group. Acupuncture treatment for new-onset IHD in PWDs was associated with a lower hazard of all-cause mortality (adjusted HR 0.78, 95% CI 0.74 to 0.81). In the group receiving the highest amount of acupuncture treatment, the hazard of mortality was the lowest, suggesting the possibility of a dose-response relationship.

In Korea, a grading system was introduced in 1988 to classify disability severity into six levels. However, starting on July 1, 2019, this system was revised to categorize disabilities as mild or severe. The Ministry of Health and Welfare of Korea classifies disabilities into 15 categories, including physical disability, brain lesion disability, visual impairment, hearing impairment, speech impairment, intellectual disability, autism spectrum disorder, mental disability, kidney disability, cardiac disability, respiratory disability, liver disability, facial disability, ostomy disability, and epilepsy. Although previous studies have shown that physical disabilities are associated with an increased risk of cardiovascular diseases, such as hypertension and coronary artery disease [9, 10, 38], this cohort study included all types of PWDs without restrictions, focusing solely on assessing the severity of disabilities. Before PSM, a comparison of baseline characteristics between the acupuncture-exposed and non-exposed groups showed that individuals with mild disabilities were more likely to use acupuncture than those with severe disabilities. PSM was performed to adjust for baseline differences between the two groups. Subgroup analysis showed a significantly lower hazard of death in both the mild and severe disability groups, with the greatest reduction observed in the high-dose acupuncture treatment group. Thus, regardless of disability severity, acupuncture treatment was associated with a lower mortality hazard. These findings suggest that acupuncture treatment could be actively recommended for PWDs diagnosed with IHD.

Disability is a complex, multidimensional concept. Traditionally, it has been viewed primarily from a medical perspective, with a focus on individuals’ physical and mental impairments. However, there has been a recent shift towards a structural and social perspective that considers environmental constraints, such as medical facilities and institutional barriers [39]. PWDs experience more unmet medical needs than non-PWDs, owing to their lower accessibility to healthcare services [40, 41]. In Korea, although the rate of unmet medical needs in PWDs is decreasing, it remains higher than that in non-PWDs. The main reasons for this disparity are the financial burden and mobility challenges [42]. Globally, efforts have been made to address the unmet medical needs of PWDs, as exemplified by Korea’s launch of a pilot project for disability primary care in May 2018. This system allows PWDs to choose one of the three types of care provided by doctors in their locality: general health management, primary disability management, or integrated management. It facilitates continuous and comprehensive care for overall health issues, including chronic diseases and disability-related conditions. Services offered include educational counseling, patient management, and home visits [43]. Participants in the pilot project emphasized the need for home visits due to mobility issues and expressed a desire for a broader range of home-based care beyond simple nursing care [44]. In Korea, medical doctors and traditional Korean medicine doctors hold equal qualifications for providing medical care. However, the system currently excludes traditional Korean medical doctors from providing multidisciplinary care, thereby limiting the healthcare choices available to PWDs. The current study found that among 134,249 PWDs diagnosed with IHD, 49,190 received acupuncture treatment, suggesting a considerable demand for traditional Korean medical treatment within this population. Furthermore, acupuncture was associated with a 22% reduction in mortality hazard, suggesting a beneficial role for acupuncture in multidisciplinary care. Therefore, it is necessary to provide traditional medical treatment options, such as acupuncture, in addition to standard care, to broaden the choices for PWDs who have unmet medical needs.

Acupuncture has long been used as an alternative therapy for IHD [45]. Patients with stable IHD who received electroacupuncture treatment at PC6 and PC4 had significantly lower risks of major cardiovascular events, recurrent unstable angina, and non-fatal myocardial infarction than those in the control group receiving standard oral medication. Differences in outcomes began to appear five months after treatment initiation, as shown by the Kaplan–Meier analysis, and multivariate Cox analysis indicated a reduction in major cardiovascular events within 12 months. Additionally, C-reactive protein levels significantly decreased after 12 months [46]. In another study, 12 weeks of acupuncture treatment in patients with stable IHD resulted in a 17% increase in vagal activity of heart rate variability compared to a sham acupuncture group, with significant increases in parasympathetic tone markers in both the time and frequency domains [47]. The primary mechanisms by which acupuncture affects IHD include increasing maximum workload and regulating blood pressure and sympathetic nervous system activity [20, 21]. Additionally, PWDs are reported to have a higher prevalence of risk factors for IHD, such as hypertension, hyperlipidemia, and diabetes [48]. Acupuncture has been shown to reduce total cholesterol, triglyceride, and low-density lipoprotein cholesterol levels [49], lower blood pressure [50], and improve fasting blood glucose and insulin resistance [51]. Thus, acupuncture may contribute to reducing mortality in PWDs diagnosed with IHD by regulating blood pressure, modulating the nervous system, and improving known risk factors. However, as previous studies have primarily focused on non-PWDs, it is unclear whether the same mechanisms apply to PWDs. Furthermore, since the pathophysiology varies by disability type, the primary causes of IHD and the mechanisms by which acupuncture exerts its effects may also differ. Therefore, further research is necessary to clarify these points.

This is the first cohort study to investigate the impact of acupuncture exposure on mortality in PWDs diagnosed with IHD, providing foundational evidence that acupuncture may contribute to reducing mortality. Additionally, the study was conducted using a large sample of PWDs diagnosed with IHD in Korea. However, this study had some limitations. A major concern is that individuals who received at least two acupuncture treatments within one year were classified as exposed, and although a reduction in mortality was observed, it is difficult to conclude that receiving just two sessions of acupuncture alone was sufficient to produce this effect. Nevertheless, the group receiving the highest number of acupuncture sessions exhibited the lowest mortality hazard, suggesting a possible dose-response relationship. As this is a cohort study, however, it inherently cannot provide definitive causal conclusions. Future verification through rigorously designed RCTs is necessary, and the effect size reported here could diminish under more controlled conditions. Second, in the dose-response analysis, the hazard did not consistently decrease with increasing acupuncture dose, instead exhibiting a J-curve pattern. This indicates possible demographic differences among groups with varying treatment frequencies, making it challenging to pinpoint an optimal dose of acupuncture. Third, exposure was defined solely by acupuncture treatment, without accounting for specific acupoints or the treatment purpose. Acupuncture is the most commonly used treatment in South Korea for musculoskeletal and connective tissue disorders [52]. However, specific acupoints are not limited to certain diseases and can have systemic effects, making it difficult to restrict acupoints [53]. Additionally, owing to the limitations of secondary data, the exact acupoints could not be identified, and only acupuncture exposure was evaluated. Fourth, the severity of IHD was not assessed. Cohort studies using NHIS data typically evaluate the medications used to determine disease severity. This study did not include such an assessment and only included patients who survived for more than one year after diagnosis, excluding those who died within the first year due to severe disease. Future research should examine how the effectiveness of acupuncture varies according to disease severity. Fifth, the subgroup analysis only confirmed disability severity and did not assess the differences in acupuncture effectiveness according to disability type. The incidence of IHD may vary according to the disability type, and certain types may respond particularly well to acupuncture treatment. Sixth, before PSM, there were significant differences in most covariates between the acupuncture and control groups. This finding suggests structural differences between the groups and the possibility of unmeasured confounding factors. Despite our efforts to adjust for various covariates, some potential confounders may still remain. Healthcare utilization behaviors, such as the frequency of outpatient visits or the proximity to medical institutions, could influence both the likelihood of receiving acupuncture and the overall survival rate. People who use healthcare services more frequently may have better-controlled chronic conditions, potentially leading to lower mortality [54]. Additionally, although we adjusted for income levels, the specific type of health insurance was not explicitly analyzed as a separate factor. In Korea, health insurance types are closely linked to socioeconomic status and can affect the accessibility and quality of medical services received by PWDs. Future studies incorporating these variables would provide a more nuanced understanding of the association between acupuncture and mortality. Finally, the appropriateness of the one-year landmark time was not explicitly validated. Although it was adopted based on previous research [18, 26–29], alternative time points were not examined. Further studies should explore whether different landmark periods affect the results.

## Conclusions

This study examined the association between acupuncture and reduced mortality in 76,314 PWDs newly diagnosed with IHD. This study evaluated whether acupuncture treatment was received within one year of IHD diagnosis and tracked mortality over three years in 38,157 individuals in both the exposure and non-exposure groups. The results showed that the group exposed to acupuncture had a significantly lower mortality hazard than the non-exposed group. Dose-response analysis revealed that the group receiving high-dose acupuncture had the lowest hazard of mortality, and subgroup analyses confirmed the robustness of the findings. Therefore, it is recommended that PWDs diagnosed with IHD receive acupuncture treatment within one year of diagnosis, regardless of the type and severity of disability.

## Supplementary Information

Supplementary Material 1.

## Data Availability

The data used in this study were obtained from the NHIS (NHIS-2021-1-301). In adherence to privacy regulations, data sharing is restricted, and the NHIS strictly prohibits the transfer, rental, or sale of databases to external parties. Researchers with approved access are eligible to request NHIS data through their official website, [https://nhiss.nhis.or.kr](https:/nhiss.nhis.or.kr) .

## Abbreviations

CCI: Charlson comorbidity index
COPD: Chronic obstructive pulmonary disease
CIs: Confidence intervals
DALYs: Disability-adjusted life years
HRs: Hazard ratios
IR: Incidence rate
IHD: Ischemic heart disease
NHIS: National health insurance service
PWDs: People with disabilities
PSM: Propensity score matching
RCT: Randomized controlled trial
SMD: Standardized mean difference
