# Supplementary material for: Association between acupuncture exposure and mortality in people with disabilities diagnosed with ischemic heart disease: a nationwide retrospective cohort study in the Republic of Korea
Source: BMC Complement Med Ther. 2026 Jun 12;26:252. doi: 10.1186/s12906-026-05434-y (PMC13491670; doi:10.1186/s12906-026-05434-y)
Supplement: Supplementary file 1 [file 12906_2026_5434_MOESM1_ESM.docx]

**Additional File 1. Definitions of covariates and clinical outcome**

| **Diagnosis** | **ICD-10-CM code and definition** | **Diagnostic definition** | |
| --- | --- | --- | --- |
| **Inclusion/exclusion criteria** |  |  | |
| Ischemic heart disease | I20, I21, I22, I23, I24, I25 |  | |
| **Intervention** |  |  | |
| Acupuncture |  | At least two times within one year after diagnosis of IHD (claim code 40011, 40012) | |
| **Charlson Comorbidity Index (Score)** |  | | |
| Myocardial infarction | I21, I22, I252 | | |
| Congestive heart failure | I099, I110, I130, I132, I255, I420, I425, I426, I427, I428, I429, I43, I50, P290 | | |
| Peripheral vascular disease | I70, I71, I731, I738, I739, I771, I790, I792, K551, K558, K559, Z958, Z959 | | |
| Cerebrovascular disease | G45, G46, I60, I61, I62, I63, I64, I65, I66, I67, I68, I69, H340 | | |
| Dementia | F00, F01, F02, F03, G30, F051, G311 | | |
| Chronic pulmonary disease | I278, I279, J40, J41, J42, J43, J44, J45, J46, J47, J60, J61, J62, J63, J64, J65, J66, J67, J684, J701, J703 | | |
| Connective tissue disease  (Rheumatologic disease) | M05, M06, M315, M32, M33, M34, M351, M353, M360 | | |
| Peptic ulcer disease | K25, K26, K27, K28 | | |
| Mild liver disease | B18, K700, K701, K702, K703, K709, K713, K714, K715, K717, K73, K74, K760, K762, K763, K764, K768, K769, Z944 | | |
| Moderate or severe liver disease  (3 points) | I850, I859, I864, I982, K704, K711, K721, K729, K765, K766, K767 | | |
| Diabetes without complications | E100, E101, E106, E108, E109, E110, E111, E116, E118, E119, E120, E121, E126, E128, E129, E130, E131, E136, E138, E139, E140, E141, E146, E148, E149 | | |
| Diabetes with complications  (2 points) | E102, E103, E104, E105, E107, E112, E113, E114, E115, E117, E122, E123, E124, E125, E127, E132, E133, E134, E135, E137, E142, E143, E144, E145, E147 | | |
| Paraplegia and hemiplegia  (2 points) | G041, G114, G800, G801,G802, G81, G82, G830, G831, G832, G833, G834, G839 | | |
| Renal disease  (2 points) | I120, I131, N030, N031, N032, N033, N034, N035, N036, N037, N038, N039, N050, N051, N052, N053, N054, N055, N056, N057, N058, N059, N18, N19, N250, Z490, Z491, Z492, Z940, Z992 | | |
| Cancer  (2 points) | C00, C01, C02, C03, C04, C05, C06, C07, C08, C09, C10, C11, C12, C13, C14, C15, C16, C17, C18, C19, C20, C21, C22, C23, C24, C25, C26, C30, C31, C32, C33, C34, C37, C38, C39, C40, C41, C43, C45, C46, C47, C48, C49, C50, C51, C52, C53, C54, C55, C56, C57, C58, C60, C61, C62, C63, C64, C65, C66, C67, C68, C69, C70, C71, C72, C73, C74, C75, C76, C81, C82, C83, C84, C85, C88, C90, C91, C92, C93, C94, C95, C96, C97 | | |
| Metastatic carcinoma  (6 points) | C77, C78, C79, C80 | | |
| AIDS/HIV  (6 points) | B20, B21, B22, B24 | | |
| **Covariates** |  |  | |
| Income | Medical aid (0 in decile)  Low (1,2,3 in decile)  Middle (4,5,6,7 in decile)  High (8,9,10 in decile) | Income Lowest 30%  Income Middle range 40%  Income Highest 30% | |
| Residential area | 1) Metropolitan  2) Urban  3) Rural | 1) The Greater Seoul Metropolitan Area (Seoul, Incheon, Gyunggi); Metropolitan area of the Republic of Korea. 26 million, more than 50% of the total population  2) Daejeon, Gwangju, Ulsan, Pusan, Daegu  3) Others | |
| Severity of disability | Severe  Mild | Grades 1 to 2  Grades 3 to 6 | |
| **Comorbidities** |  | | |
| Hypertension | I10, I11, I12, I13, I15 | | Admission ≥1 or outpatient department ≥2 |
| Diabetes mellitus | E10-E14 | | Admission ≥1 or outpatient department ≥2 |
| Dyslipidemia | E78 | | Admission ≥1 or outpatient department ≥2 |
| Ischemic stroke | I63 | | Admission ≥1 or outpatient department ≥2 along with the presence of a brain CT or MRI (Brain CT: HA441, HA451, HA461, HA531 or Brain MRI: HF101, HI101, HI135, HI201). |
| COPD | J41-J44 | | Admission ≥1 or outpatient department ≥2 |
| Atrial fibrillation | I48 | | Admission ≥1 or outpatient department ≥2 |
| Peripheral artery disease | I70,I73 | | Admission ≥1 or outpatient department ≥2 |
| Chronic kidney disease | N18, N19 | | Admission ≥1 or outpatient department ≥2 |
| Chronic liver disease | B18, K70-K74, K76.1 | | Admission ≥1 or outpatient department ≥2 |
| **Clinical outcome** |  |  | |
| All-cause mortality |  |  | |

**AIDS, acquired immunodeficiency syndrome; COPD, chronic obstructive pulmonary disease; HIV, human immunodeficiency virus; CT, computed tomography; ICD-10-CM, International Classification of Diseases, 10th Revision, Clinical Modification; IHD, ischemic heart disease; MRI, magnetic resonance imaging.**

**Additional File 2. Forest plots for subgroup analysis**

**AF, atrial fibrillation; CCI, Charlson Comorbidity Index; COPD, chronic obstructive pulmonary disease; DM, diabetes mellitus; HTN, hypertension.**

The conventional group comprised individuals who did not receive acupuncture. Those who received acupuncture were grouped by the number of sessions, from the lowest dose in the acupuncture 1 group to the highest dose in the acupuncture 4 group.
